# Comparison of Long-term Survival of Patients With Early-Stage Non–Small Cell Lung Cancer After Surgery vs Stereotactic Body Radiotherapy

**DOI:** 10.1001/jamanetworkopen.2019.15724

**Published:** 2019-11-20

**Authors:** Alexander Chi, Wei Fang, Yeping Sun, Sijin Wen

**Affiliations:** 1 Marshfield Clinic, Marshfield, Wisconsin; 2 West Virginia Clinical and Translational Science Institute, Erma Byrd Biomedical Research Center, West Virginia University Health Sciences Center, Morgantown; 3 Department of Biostatistics, West Virginia University Health Sciences Center, Morgantown

## Abstract

**Importance:**

Previous comparisons of surgery and stereotactic body radiotherapy (SBRT) for early-stage (ES) non–small cell lung cancer (NSCLC) did not account for the extent of regional lymph node examination (LNE) during surgery.

**Objective:**

To compare long-term overall survival (OS) of patients with ES NSCLC after surgery vs SBRT when the extent of regional LNE in patients undergoing surgery is thoroughly considered.

**Design, Setting, and Participants:**

Cohort study with survival comparisons using the multivariable Cox proportional hazards model and after propensity score matching. Data from the National Cancer Database were analyzed from October 28, 2018, through April 18, 2019. Patients with ES NSCLC diagnosed between January 1, 2004, and December 31, 2015, who underwent any curative-intent surgery or SBRT were included.

**Main Outcomes and Measures:**

Long-term OS.

**Results:**

Of 104 709 total patients, 91 330 underwent surgery (42 508 [46.5%] male; median [interquartile range] age, 68 [61-75] years) and 13 379 received SBRT (6065 [45.3%] male; median [interquartile range] age, 75 [68-81] years). Surgery, especially lobectomy (hazard ratio [HR], 0.53; 95% CI, 0.50-0.56), and regional LNE, especially when more than 10 lymph nodes were examined (HR, 0.73; 95% CI, 0.69-0.77), were associated with better long-term OS (*P* < .001). Pneumonectomy was not associated with reduced mortality risk when 0 nodes were examined (HR for stage T1, 1.43; 95% CI, 0.67-3.06; *P* = .35; HR for stage T2-T3, 0.62; 95% CI, 0.34-1.13; *P* = .12) or when more than 15 nodes were examined for stage T1 disease in patients younger than 80 years (HR, 0.77; 95% CI, 0.54-1.09; *P* = .14) or when patients aged 80 years or older received regional LNE of any extent (>15 nodes examined: HR for stage T1, 0.65; 95% CI, 0.16-2.64; *P* = .54; HR for stage T2-T3, 0.90; 95% CI, 0.50-1.60; *P* = .71). Less extensive surgery was not associated with improved OS when 0 nodes were examined in patients aged 80 years or older with stage T2 to T3 tumors (HR for lobectomy, 0.90; 95% CI, 0.65-1.25; *P* = .53) and in selected operable patients older than 75 years with stage T1 tumors (HR for lobectomy, 1.07; 95% CI, 0.57-2.00; *P* = .84).

**Conclusions and Relevance:**

This study found that, overall, surgery coupled with regional LNE of appropriate extent was associated with the best long-term OS in patients with ES NSCLC.

## Key Points

**Question:**

How does the long-term survival after curative-intent surgery with regional lymph node examination of various extents compare with long-term survival after stereotactic body radiotherapy for early-stage non–small cell lung cancer?

**Findings:**

In this cohort study of 104 709 patients in the US National Cancer Database with early-stage non–small cell lung cancer, those who received surgery coupled with regional lymph node examination of an appropriate extent had significantly better long-term survival than those who received stereotactic body radiotherapy.

**Meaning:**

These findings suggest that curative-intent surgery, when coupled with regional lymph node examination, is generally associated with the best long-term overall survival in patients with early-stage non–small cell lung cancer.

## Teaser

This cohort study compares long-term overall survival of patients with early-stage non–small cell lung cancer treated with stereotactic body radiotherapy vs surgery including various degrees of lymph node examination.

## Introduction

Lung cancer is the leading cause of cancer-related death in the United States and worldwide, with non–small cell lung cancer (NSCLC) accounting for more than 80% of all cases diagnosed.^1,2,3^ The standard curative treatment for early-stage (ES) NSCLC (stage I or II) is lobectomy combined with systematic or lobe-specific lymph node dissection and/or sampling, although other options of anatomic pulmonary resection and noninvasive treatments are also available.^3,4,5,6,7^ Owing to the prognostic and therapeutic implications of regional nodal metastasis, accurate lymph node staging based on the standard of care is of critical importance during surgery for ES NSCLC.^7,8,9^ However, the number of regional lymph nodes examined during lung cancer surgery is highly variable in daily clinical practice. Many patients who underwent lobar or greater resection had no lymph node examination (LNE), fewer than 6 lymph nodes examined, or inadequate retrieval of intrapulmonary lymph nodes from resection specimens.^9,10,11,12,13,14^ All led to understaging and worse-than-expected stage-stratified survival, while the most optimal number of regional lymph nodes to be examined may be well beyond what was recommended.^11,14^

Heterogeneity in stage-stratified survival in pathologically N0 NSCLC due to variations in the extent of regional LNE imposes a challenge in the comparison of surgery with other noninvasive therapies, such as stereotactic body radiotherapy (SBRT), for ES NSCLC. In SBRT, an ablative dose of radiation is delivered to the primary tumor over several daily fractions. It has been associated with excellent local control in ES NSCLC.^15,16^ However, how it compares with surgery in patients with ES NSCLC remains controversial, with conflicting results being reported.^17,18,19,20,21,22,23,24,25,26,27^ One reason may be that the extent of regional LNE during surgery has not been fully accounted for in previous comparisons. In this study, we compare the long-term overall survival (OS) following curative-intent surgery with OS following SBRT for ES NSCLC, with the extent of regional LNE in patients undergoing surgery thoroughly considered.

## Methods

### Data Source

We used data from the National Cancer Database for patients with lung cancer diagnosed between 2004 and 2015. This was the most recent data set available at the time of the study. The National Cancer Database is a joint project of the Commission on Cancer of the American College of Surgeons and the American Cancer Society. This hospital-based, nationwide database captures approximately 70% of incident cancer cases in the United States. It provides deidentified data subsets to investigators from Commission on Cancer–accredited programs through an online application process. The study was exempt from review by the institutional review board of the Marshfield Clinic. Informed consent was waived as there was no increased harm to patients owing to the study’s retrospective nature. This study followed the Strengthening the Reporting of Observational Studies in Epidemiology (STROBE) reporting guideline.

### Study Cohort

First, patients with primary NSCLC and no prior diagnosis of any malignant neoplasm were selected from the requested data set. This study cohort was limited to patients with histological diagnosis of invasive cancer who underwent treatment in a Commission on Cancer facility. Next, patients with clinical stage of T1 to T3, N0, and M0, as defined by the American Joint Committee on Cancer *AJCC Staging Manual, 8th Edition *staging criteria, were selected based on clinical TNM staging information and tumor size. From this study population, we created a surgery cohort and a SBRT cohort.

**Figure 1.**
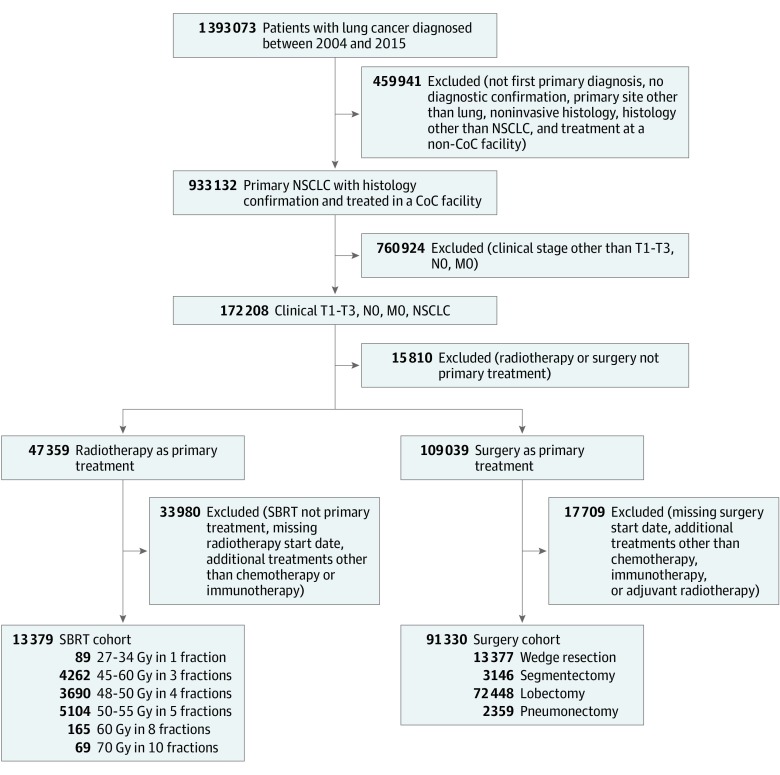
Flow Diagram for Patient Selection Clinical stage T1 to T3, N0, M0 non–small cell lung cancer (NSCLC) was determined according to the criteria of the American Joint Committee on Cancer *AJCC Staging Manual, 8th Edition*. CoC indicates Commission on Cancer; SBRT, stereotactic body radiotherapy.

For the surgery cohort, only patients who underwent wedge resection, segmentectomy, lobectomy, or pneumonectomy were included. Neoadjuvant or adjuvant systemic therapy (chemotherapy or immunotherapy) or adjuvant radiotherapy was allowed. Patients with missing data on surgery starting time and the starting time for other treatments were excluded. For the SBRT cohort, patients who received primary photon or proton beam irradiation over 1 to 10 fractions to the thorax were selected. Patients with missing information regarding treatment start or end days from diagnosis were excluded. Patients who received additional treatments other than chemotherapy or immunotherapy or with unknown information on treatments other than radiotherapy were excluded. Only patients who were treated with dose fraction schedules per National Comprehensive Cancer Network guidelines that are equivalent to a biologically effective dose of at least 100 Gy_10_ (Gy calculated with an α to β ratio of 10 Gy) were included. Biologically effective dose was calculated as total dose × [1 + fractional dose / (α / β)]. These included 27 to 34 Gy in 1 fraction, 45 to 60 Gy in 3 fractions, 48 to 50 Gy in 4 fractions, 50 to 55 Gy in 5 fractions, 60 Gy in 8 fractions, and 70 Gy in 10 fractions (Figure 1).

### Study Variables

Patient, tumor, and treatment characteristics were provided by the National Cancer Database or derived from the variables provided. They include year of diagnosis, age, sex, race, Charlson Comorbidity Index score, anatomical tumor location, tumor histology, tumor size, clinical T stage, regional LNE (the total number of regional lymph nodes removed and examined) in patients who underwent surgery, tumor extension, nodule plurality in the ipsilateral lung, systemic therapy, insurance, urban vs rural status, income quartile, education quartile, and facility type, location, and volume.

### Outcome

The primary end point was OS, which was measured from the time of diagnosis to date of death from any cause or last follow-up. Patients still alive or without any clear indication of death at last follow up were censored at that point.

### Statistical Analysis

The data were analyzed from October 28, 2018, through April 18, 2019. Descriptive statistics were used to summarize patients’ baseline and clinical characteristics. Categorical data were described using contingency tables including counts and percentages. Continuously scaled measures were summarized with descriptive statistical measures (ie, mean [standard deviation] or median [range]). Independence between categorical variables was assessed with the χ^2^ test. Differences between the 2 groups in continuous variables were assessed with the Wilcoxon rank sum test.

Overall survival was estimated using the Kaplan-Meier method, and compared between the SBRT and surgery cohorts using the log-rank test in a univariate analysis. In the multivariate analysis, the Cox proportional hazards regression model stratified by tumor grade was used to compare survival following different treatments while adjusting for all major known variables (listed in the Study Variables subsection of Methods). The proportional hazards assumption was assessed with the Grambsch-Therneau test.^28^ The stratified Cox model was fitted if a variable associated with OS did not satisfy the proportional hazards assumption.

Next, stratified multivariable Cox models were used to compare surgery and SBRT in more homogeneous populations stratified by independent variables, such as age, T stage, and the extent of regional LNE (patients who underwent surgery only). In particular, a grade-stratified multivariable Cox model that adjusted for potential confounders was used to compare treatments in each stratum.

Propensity score matching was also performed to compare surgery and SBRT incorporating preoperative risk factors significantly associated with OS. Sublobar resection (wedge resection or segmentectomy) and lobar resection (lobectomy or pneumonectomy) were compared with SBRT separately after propensity score matching. Propensity scores were calculated using logistic regression with treatment as the dependent variable. Patients were matched 1:1 using our preliminary propensity score model in which the estimand is the mean treatment effect on SBRT.^29^ Success of propensity score matching was assessed by measuring the balance of the confounders for every matching variable.

All statistical tests were 2-sided and *P* < .05 was considered statistically significant. Statistical analyses were carried out using SAS statistical software version 9.1 (SAS Institute) and R statistical software version 3.0.3 (R Project for Statistical Computing) package “Matching.”^29^

## Results

### Study Cohort Characteristics

Of 104 709 total patients, 91 330 (42 508 [46.5%] male; median [interquartile range] age, 68 [61-75] years) were included in the surgery cohort, and 13 379 (6065 [45.3%] male; median [interquartile range] age, 75 [68-81] years) were included in the SBRT cohort. Most patients who underwent surgery received either lobectomy (79.3%) or wedge resection (14.7%). Major baseline patient characteristics are listed in Table 1. Receiving SBRT was associated with older age, lower clinical T stage, adenocarcinoma histology, tumor confinement to 1 lung, and single tumor nodule. Examination of 1 to 10, 11 to 15, and more than 15 regional lymph nodes was conducted in 55.8%, 15.6%, and 14.2% of patients, respectively, in the surgery cohort. Most patients did not receive any systemic therapy as part of their primary treatment (eTable 1 in the Supplement). Regional LNE of limited extent and regional LN aspiration or biopsy were performed in 4.21% and 3.92% of the patients in the SBRT cohort, respectively. In the SBRT cohort, regional LNE of limited extent (HR vs SBRT alone, 1.10; 95% CI, 0.96-1.28; *P* = .17) or regional LN aspiration or biopsy (HR vs SBRT alone, 0.99; 95% CI, 0.97-1.02; *P* = .69) were not associated with any significant difference in OS when compared with SBRT alone.

**Table 1.** Patient Characteristics

Characteristic	No. (%)		*P* Value
	SBRT (n = 13 379)	Surgery (n = 91 330)	
Age, median (IQR), y	75 (68-81)	68 (61-75)	<.001
Year of diagnosis			
2004-2009	1974 (14.8)	26 730 (29.3)	<.001
2010-2015	11 405 (85.2)	64 600 (70.7)	
Sex			
Male	6065 (45.3)	42 508 (46.5)	.009
Female	7314 (54.7)	48 822 (53.5)	
Race			
White	11 887 (88.9)	80 521 (88.2)	<.001
Black	1180 (8.8)	7423 (8.1)	
Other	215 (1.6)	2789 (3.1)	
Charlson Comorbidity Index score			
0	6990 (52.3)	44 097 (48.3)	<.001
1	3818 (28.5)	32 968 (36.1)	
2 or 3	2571 (19.2)	14 265 (15.6)	
Grade			
Well differentiated	1078 (8.1)	14 249 (15.6)	<.001
Moderately differentiated	2448 (18.3)	41 726 (45.7)	
Poorly differentiated or undifferentiated	2826 (21.1)	30 501 (33.4)	
Unknown	7027 (52.5)	4854 (5.3)	
Histology			
Adenocarcinoma	6522 (48.8)	58 070 (63.6)	<.001
Squamous cell carcinoma	5143 (38.4)	27 359 (30.0)	
Other	1714 (12.8)	5901 (6.5)	
Anatomical site			
Right upper lobe	4448 (33.3)	31 631 (34.6)	<.001
Right middle lobe	589 (4.4)	4442 (4.9)	
Right lower lobe	2339 (17.5)	16 302 (17.9)	
Left upper lobe	3768 (28.2)	23 542 (25.8)	
Left lower lobe	1940 (14.5)	12 779 (14.0)	
Overlapping	30 (0.2)	938 (1.0)	
Lung, not otherwise specified	214 (1.6)	1247 (1.4)	
Other	51 (0.4)	449 (0.5)	
Clinical T stage			
T1a	487 (3.6)	5759 (6.3)	<.001
T1b	4706 (35.2)	28 647 (31.4)	
T1c	4149 (31.0)	20 178 (22.1)	
T2a	2456 (18.4)	19 969 (21.9)	
T2b	794 (5.9)	6907 (7.6)	
T3	664 (5.0)	9706 (10.6)	
Other	123 (0.9)	164 (0.2)	
Tumor extension			
Confined to 1 lung	9257 (69.2)	49 026 (53.7)	<.001
Adjacent lobe extension	20 (0.2)	205 (0.2)	
Central location	121 (0.9)	2693 (3.0)	
Pleura, chest wall, or diaphragm	289 (2.2)	14 061 (15.4)	
Atelectasis or obstructive pneumonia	149 (1.1)	929 (1.0)	
Unknown	3543 (26.5)	24 416 (26.7)	
Separate nodules in the ipsilateral lung			
None	10 854 (81.1)	61 160 (67.0)	<.001
Same lobe	297 (2.2)	2622 (2.9)	
Different lobes	0	0	
Unknown	2228 (16.7)	27 548 (30.2)	
Scope of lymph node surgery			
No	12 750 (95.3)	7437 (8.1)	<.001
Yes	599 (4.5)	83 573 (91.5)	
Unknown	30 (0.2)	320 (0.4)	
Regional lymph nodes examined, No.			
0	12 238 (91.5)	7382 (8.1)	<.001
1-10	371 (2.8)	50 977 (55.8)	
11-15	17 (0.1)	14 256 (15.6)	
>15	14 (0.1)	12 944 (14.2)	
Other	739 (5.5)	5771 (6.3)	

Abbreviations: IQR, interquartile range; SBRT, stereotactic body radiotherapy.

### Survival Analysis

**Figure 2.**
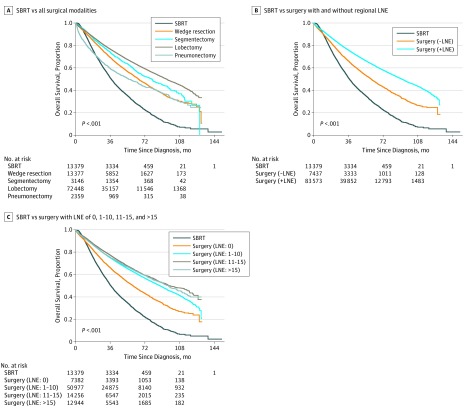
Overall Survival for Stereotactic Body Radiotherapy (SBRT) vs Surgery LNE indicates lymph node examination.

Patient survival stratified by treatment approach is shown in Figure 2A. The unadjusted 5-year OS was 48.1% to 64.6% in the surgery cohort depending on the type of surgery performed and 30.4% in the SBRT cohort (HR for wedge resection vs SBRT, 0.55; 95% CI, 0.52-0.57; *P* < .001; HR for segmentectomy vs SBRT, 0.48; 95% CI, 0.43-0.49; *P* < .001; HR for lobectomy vs SBRT, 0.40; 95% CI, 0.39-0.42; *P* < .001; HR for pneumonectomy vs SBRT, 0.71; 95% CI, 0.67-0.76; *P* < .001). The improved long-term OS associated with surgery compared with SBRT was augmented by the conduct of regional lymph node surgery and increased number of regional lymph nodes examined (Figure 2B and C). In patients who underwent surgery at the primary tumor site, the conduct of regional lymph node surgery was associated with better 5-year OS (63.6% vs 49.8%; HR vs no lymph node surgery, 0.66; 95% CI, 0.64-0.69; *P* < .001). For patients who underwent surgery with 0, 1 to 10, 11 to 15, and more than 15 lymph nodes examined, 5-year OS was 50.2%, 62.9%, 65.3%, and 64.6%, respectively (HR for 1-10 lymph nodes examined vs 0, 0.68; 95% CI, 0.66-0.71; *P* < .001; HR for 11-15 lymph nodes examined vs 0, 0.62; 95% CI, 0.59-0.64; *P* < .001; HR for >15 lymph nodes examined vs 0, 0.64; 95% CI, 0.61-0.68; *P* < .001).

Primary treatment and the number of regional lymph nodes examined along with all baseline characteristics, except chemotherapy and immunotherapy as part of the primary treatment, were significantly associated with survival in a univariate analysis (eTable 2 in the Supplement). After stratifying for tumor grade and adjusting for all variables that may affect the entire patient population’s prognosis in a multivariate Cox model (Table 2), surgical treatments were found to be associated with a reduction in mortality risk over SBRT (wedge resection: HR, 0.67; 95% CI, 0.64-0.71; *P* < .001; segmentectomy: HR, 0.60; 95% CI, 0.56-0.65; *P* < .001; lobectomy: HR, 0.53; 95% CI, 0.50-0.56; *P* < .001; pneumonectomy: HR, 0.75; 95% CI, 0.69-0.82; *P* < .001). For the extent of regional LNE as an independent variable, examination of more than 10 lymph nodes was associated with the greatest reduction in mortality risk when compared with no LNE (11-15 lymph nodes examined: HR, 0.73; 95% CI, 0.69-0.77; *P* < .001; >15 lymph nodes examined: HR, 0.73; 95% CI, 0.69-0.77; *P* < .001) in the entire patient population. In the Cox model, other variables that were associated with a reduction in mortality risk were younger age, lower Charlson Comorbidity Index score, adenocarcinoma histology, nonoverlapping location, tumor limited to the lung parenchyma, and solitary tumor nodule.

**Table 2.** Mortality Risk Based on Independent Variables Associated With Survival

Study Variables	Univariate Analysis *P* Value	HR (95% CI)^a^	*P* Value
Age, y			
≤65	<.001	1 [Reference]	
66-70		1.18 (1.14-1.22)	<.001
71-75		1.38 (1.33-1.43)	<.001
76-80		1.68 (1.62-1.75)	<.001
>80		1.90 (1.82-1.98)	<.001
Charlson Comorbidity Index score			
0	<.001	1 [Reference]	
1		1.18 (1.15-1.21)	<.001
2 or 3		1.48 (1.44-1.53)	<.001
Histology			
Adenocarcinoma	<.001	1 [Reference]	
Squamous cell carcinoma		1.12 (1.09-1.14)	<.001
Adenosquamous		1.38 (1.30-1.47)	<.001
Large cell carcinoma		1.20 (1.10-1.31)	<.001
Other (lymphoepithelioma, undifferentiated non–small cell lung cancer)		1.15 (1.09-1.21)	<.001
Tumor location			
Right upper lobe	<.001	1 [Reference]	
Right middle lobe		1.07 (1.01-1.13)	.02
Right lower lobe		1.11 (1.08-1.15)	<.001
Left upper lobe		1.01 (0.98-1.04)	.39
Left lower lobe		1.02 (0.98-1.06)	.30
Overlapping		1.23 (1.11-1.37)	<.001
Lung, not otherwise specified		1.17 (1.07-1.28)	.001
Other		1.00 (0.86-1.17)	.96
T stage			
T1	<.001	1 [Reference]	
T2		1.13 (1.09-1.19)	<.001
T3		1.36 (1.26-1.47)	<.001
Tumor extension			
Confined to 1 lung	<.001	1 [Reference]	
Adjacent lobe extension		1.50 (1.16-1.93)	.002
Mainstem bronchus, carina, or hilum		1.05 (0.98-1.12)	.17
Other central structures		1.52 (1.32-1.73)	<.001
Pleura, chest wall, or diaphragm		1.35 (1.30-1.40)	<.001
Atelectasis or obstructive pneumonia		1.13 (1.02-1.25)	.02
Separate nodules, ipsilateral lung			
None	<.001	1 [Reference]	
Yes			
Same lobe		1.13 (1.04-1.22)	.004
Different lobe involved		1.32 (1.14-1.53)	<.001
Separate tumor nodules, not otherwise specified		1.52 (1.02-2.25)	.04
Not available or unknown		1.26 (1.07-1.49)	.005
Regional lymph nodes examined, No.			
0	<.001	1 [Reference]	
1-10		0.82 (0.78-0.85)	<.001
11-15		0.73 (0.69-0.77)	<.001
>15		0.73 (0.69-0.77)	<.001
Treatment			
Stereotactic body radiotherapy	<.001	1 [Reference]	
Wedge resection		0.67 (0.64-0.71)	<.001
Segmentectomy		0.60 (0.56-0.65)	<.001
Lobectomy		0.53 (0.50-0.56)	<.001
Pneumonectomy		0.75 (0.69-0.82)	<.001

Abbreviation: HR, hazard ratio.

^a^ Also adjusted for sex, race, tumor size, systemic therapy, insurance, urban/rural, income quartile, education quartile, facility type, facility location, and facility volume quartile.

### Survival Comparisons in Stratified Analyses Using the Multivariable Cox Model

Survival after surgery was also compared with survival after SBRT in 16 patient population partitions that were stratified by age, clinical T stage, and then the extent of regional LNE in surgery patients. A stratified multivariable Cox model was used to compare OS after surgery with OS after SBRT adjusting for all potential confounding variables. Overall, surgery was associated with a reduction in mortality risk over SBRT. Reduction in mortality risk became more obvious as more regional lymph nodes were examined (eFigure in the Supplement). In patients younger than 80 years, sublobar resections and lobectomy with regional LNE of any extent were associated with a reduction in mortality risk over SBRT (>15 nodes examined: HR for stage T1, 0.65; 95% CI, 0.16-2.64; *P* = .54; HR for stage T2-T3, 0.90; 95% CI, 0.50-1.60; *P* = .71) (Table 3). Pneumonectomy was not associated with any reduction in mortality risk over SBRT when it was coupled with no regional LNE (for stage T1: HR, 1.43; 95% CI, 0.67-3.06; *P* = .35; for stage T2-T3: HR, 0.62; 95% CI, 0.34-1.13; *P* = .12) or LNE of more than 15 lymph nodes if patients had stage T1 tumors (HR, 0.77; 95% CI, 0.54-1.09; *P* = .14). In patients aged 80 years or older, pneumonectomy with regional LNE of any extent was not associated with any reduction in mortality risk over SBRT (Table 3). Sublobar resection (mainly wedge resection) and lobectomy were generally associated with a reduction in mortality risk over SBRT, except in patients with stage T2 to T3 tumors who did not undergo any regional LNE (wedge resection: HR, 0.81; 95% CI, 0.64-1.02; *P* = .07; segmentectomy: HR, 1.15; 95% CI, 0.65-2.05; *P* = .64; lobectomy: HR, 0.90; 95% CI, 0.65-1.25; *P* = .53).

**Table 3.** Age- and T Stage–Dependent Mortality Risk Associated With Surgery vs SBRT in 16 Patient-Population Partitions

Treatment	Age, y			
	<80		≥80	
	HR (95% CI)^a^	*P* Value	HR (95% CI)^a^	*P* Value
**Stage T1**				
LNE: 0				
No.	10 986		3638	
SBRT	1 [Reference]		1 [Reference]	
Wedge resection	0.62 (0.57-0.67)	<.001	0.83 (0.73-0.95)	.006
Segmentectomy	0.51 (0.41-0.63)	<.001	0.73 (0.51-1.05)	.09
Lobectomy	0.47 (0.41-0.54)	<.001	0.68 (0.51-0.91)	.008
Pneumonectomy	1.43 (0.67-3.06)	.35		
LNE: 1-10				
No.	35 109		5459	
SBRT	1 [Reference]		1 [Reference]	
Wedge resection	0.47 (0.44-0.51)	<.001	0.62 (0.54-0.72)	<.001
Segmentectomy	0.39 (0.35-0.45)	<.001	0.68 (0.54-0.87)	.002
Lobectomy	0.35 (0.33-0.38)	<.001	0.52 (0.47-0.58)	<.001
Pneumonectomy	0.63 (0.49-0.81)	<.001	1.75 (0.65-4.71)	.27
LNE: 11-15				
No.	13 477		3304	
SBRT	1 [Reference]		1 [Reference]	
Wedge resection	0.41 (0.33-0.51)	<.001	0.38 (0.21-0.66)	<.001
Segmentectomy	0.40 (0.28-0.57)	<.001	0.26 (0.06-1.03)	.06
Lobectomy	0.32 (0.30-0.35)	<.001	0.47 (0.39-0.56)	<.001
Pneumonectomy	0.66 (0.45-0.94)	.02	1.03 (0.25-4.30)	.97
LNE: >15				
No.	12 032		3248	
SBRT	1 [Reference]		1 [Reference]	
Wedge resection	0.34 (0.26-0.46)	<.001	0.31 (0.13-0.75)	.01
Segmentectomy	0.29 (0.18-0.46)	<.001	0.86 (0.42-1.75)	.67
Lobectomy	0.33 (0.30-0.37)	<.001	0.46 (0.38-0.55)	<.001
Pneumonectomy	0.77 (0.54-1.09)	.14	0.65 (0.16-2.64)	.54
**Stage T2-T3**				
LNE: 0				
No.	3770		1628	
SBRT	1 [Reference]		1 [Reference]	
Wedge resection	0.62 (0.54-0.71)	<.001	0.81 (0.64-1.02)	.07
Segmentectomy	0.66 (0.49-0.88)	.005	1.15 (0.65-2.05)	.64
Lobectomy	0.41 (0.35-0.49)	<.001	0.90 (0.65-1.25)	.53
Pneumonectomy	0.62 (0.34-1.13)	.12	3.07 (0.66-14.15)	.15
LNE: 1-10				
No.	19 584		3465	
SBRT	1 [Reference]		1 [Reference]	
Wedge resection	0.52 (0.47-0.59)	<.001	0.63 (0.51-0.78)	<.001
Segmentectomy	0.51 (0.43-0.59)	<.001	0.49 (0.35-0.68)	<.001
Lobectomy	0.39 (0.36-0.42)	<.001	0.57 (0.50-0.65)	<.001
Pneumonectomy	0.50 (0.43-0.58)	<.001	0.90 (0.57-1.42)	.65
LNE: 11-15				
No.	8206		1909	
SBRT	1 [Reference]		1 [Reference]	
Wedge resection	0.47 (0.34-0.66)	<.001	0.50 (0.22-1.15)	.10
Segmentectomy	0.36 (0.22-0.57)	<.001	0.46 (0.20-1.05)	.06
Lobectomy	0.33 (0.30-0.37)	<.001	0.53 (0.44-0.63)	<.001
Pneumonectomy	0.47 (0.39-0.56)	<.001	1.17 (0.42-3.25)	.77
LNE: >15				
No.	8333		1971	
SBRT	1 [Reference]		1 [Reference]	
Wedge resection	0.37 (0.25-0.55)	<.001	0.32 (0.12-0.84)	.02
Segmentectomy	0.35 (0.20-0.59)	<.001	0.61 (0.04-10.50)	.73
Lobectomy	0.38 (0.34-0.42)	<.001	0.54 (0.45-0.64)	<.001
Pneumonectomy	0.49 (0.42-0.58)	<.001	0.90 (0.50-1.60)	.71

Abbreviations: HR, hazard ratio; LNE, lymph node examination; SBRT, stereotactic body radiotherapy.

^a^ Adjusted for all potential confounding variables.

Treatments were further compared in patients older than 75 years with Charlson Comorbidity Index score of 0 and stage T1 tumors after adjusting for other variables, including the extent of regional LNE in patients who underwent surgery (eTable 3 in the Supplement). Only patients in the SBRT cohort who were operable but refused surgery and received a dose at least biologically equivalent to 50 Gy delivered in 4 fractions were included. In this subpopulation, sublobar resection (HR, 1.17; 95% CI, 0.64-2.15; *P* = .60) and lobectomy (HR, 1.07; 95% CI, 0.57-2.00; *P* = .84) were not associated with any reduction in mortality risk, while pneumonectomy was associated with an increase in mortality risk over SBRT (HR, 3.20; 95% CI, 1.20-8.49; *P* = .02).

### Survival Comparison in Propensity Score–Matched Cohorts

For the propensity score–matched cohorts, the balance for each variable indicates good balance between 12 632 patients undergoing sublobar resection and 12 632 patients undergoing SBRT, as well as 12 632 patients undergoing lobar resection and 12 632 patients undergoing SBRT (eTable 4 and eTable 5 in the Supplement). Both sublobar resection (HR, 0.56; 95% CI, 0.54-0.58, *P* < .001) and lobar resection (HR, 0.47; 95% CI, 0.45-0.49, *P* < .001) were associated with a reduction in mortality risk compared with SBRT.

## Discussion

Overall, all surgical modalities studied were associated with superior long-term OS when compared with SBRT in patients with clinical stage T1 to T3, N0, M0 NSCLC (Figure 2A). This survival advantage is further enhanced by regional LNE, especially when more than 10 lymph nodes were examined (Figure 2B and C). Although surgery’s superiority over SBRT has been corroborated in many studies,^17,18,19,20,21,22,23^ the influence of regional lymph node assessment on such comparisons has not, to our knowledge, been fully analyzed previously. Regional LNE is an important factor to consider when comparing surgery and SBRT for ES NSCLC owing to its association with stage-stratified survival after surgery for NSCLC.^9,10,11,12,13,14^ In the current study, the survival advantage associated with surgery over SBRT remained after adjusting for the number of regional lymph nodes examined and other known variables in a multivariable Cox model (Table 2). Among all surgical modalities, lobectomy was associated with the lowest mortality risk. Regional LNE was independently associated with survival, and examination of more than 10 lymph nodes was associated with the lowest mortality risk. Surgery’s association with a reduction in mortality risk over SBRT, especially when coupled with regional LNE, was also demonstrated in a comparison after propensity score matching (eTable 4 and eTable 5 in the Supplement). Overall, our findings are consistent with previous studies and the currently guidelines, which support lobectomy with adequate regional lymph node assessment to be the standard of care in operable patients with ES NSCLC.^3,4,5,17,18,19,20,21,22,23^

At least comparable OS following surgery and SBRT has been suggested in some studies.^24,25,26,27^ However, thorough regional LNE of an adequate number of lymph nodes during surgery might not have been routinely performed in these studies, leading to underestimation of long-term OS after surgery. For instance, only 37% to 71.9% of the patients had 6 or more lymph nodes dissected during surgery in 2 propensity-matched studies that found similar survival following lobectomy and SBRT for ES NSCLC.^24,25^ In an analysis of the Surveillance, Epidemiology, and End Results–Medicare database, similar 3-year OS and lung cancer–specific survival were observed between lobectomy and SBRT after propensity matching of 502 patients with ES NSCLC.^26^ In this study, the only known regional lymph node assessment conducted was mediastinal lymph node sampling, which was done in only 8% of the propensity score–matched patients. Although more thorough regional LNE was required for patients who underwent lobectomy in a pooled study of limited sample size, no details on the number of nodal stations and lymph nodes examined during surgery were reported.^27^

How surgery coupled with regional LNE compares with SBRT appeared to be associated with patient age, T stage, and the extent of surgery for both the primary tumor and regional lymph nodes. Comparable survival between surgery and SBRT may be observed in some situations. In further analysis of more homogeneous population partitions, pneumonectomy was not associated with lower mortality risk over SBRT regardless of whether regional lymph nodes were examined in patients aged 80 years and older. In patients younger than 80 years, pneumonectomy was associated with a significant reduction in mortality risk over SBRT only when coupled with regional LNE. However, this only occurred when fewer than 16 lymph nodes were examined if patients had T1 tumors. Surgery is known to be associated with a higher incidence of morbidity and mortality following treatment compared with SBRT.^27,30^ This difference in mortality after treatment appears to increase with age, while postoperative mortality also increases with more extensive surgery.^30^ Thus, very extensive surgery at either the primary site or regional nodal stations may lead to higher-than-expected risk for postoperative morbidity and mortality. This may be more prominent in older patients, as they are less likely to tolerate surgery owing to increased likelihood of frailty and comorbidities. As a result, SBRT may be a reasonable alternative treatment in these situations. For the same reasons, SBRT appears to be associated with better shorter-term survival over surgery, especially pneumonectomy (Figure 2).

Surgery was associated with a significant reduction in mortality risk over SBRT in patients with T2 to T3 tumors who were aged 80 years and older only when regional lymph nodes were examined. The risk of regional lymph node involvement increases as T stage increases in NSCLC,^31,32^ which makes thorough regional LNE more critical for ES NSCLC with stage greater than T1. However, it is unlikely to be the predominant reason in this situation, which was not observed in patients younger than 80 years. This suggests a strong influence from life expectancy when comparing OS following surgery and SBRT in patients with ES NSCLC, because older patients are more likely to die of noncancer causes,^18,21^ including postoperative morbidity. Second, patients with T2 to T3 tumors who underwent surgery but not regional LNE may have been thought unlikely to benefit from it or unable to tolerate it, which is more likely in patients aged 80 years or older. In this situation, SBRT may be a good alternative to consider over surgery.

To investigate how surgery compares with SBRT in older patients with longer life expectancy and a relatively lower risk for regional lymph node metastasis based on T stage, further analysis was conducted in a selected group of operable patients older than 75 years with excellent comorbidity score and T1 tumors. To ensure adequate radiation dosing, only patients who received a higher-than-accepted-minimum dose were chosen if they underwent SBRT based on a previous study.^27^ Our findings suggest that similar OS may be observed after surgery and SBRT in operable healthy patients with small NSCLC tumors. Such similarity was lost when patients with T2a tumors were included in this subgroup analysis, with superiority associated with lobectomy becoming significant. Our observation may be related to a lower risk of regional lymph node metastasis in T1 vs T2 to T3 tumors. However, this remains to be further investigated.

### Limitations

This study has limitations. Unlike randomized clinical trials, cohort studies are known to be vulnerable to selection bias and confounding due to both known and unknown factors.^33^ As a result, validity of any conclusion from cohort studies is often questioned with great concern. Many methods exist to minimize selection bias and confounding, thus increasing the internal validity of a cohort study comparing different treatments.^34,35^ In this study, we used the Cox proportional hazards regression model to account for all available variables that may be of prognostic value to the entire study population and conducted comparison analyses after propensity score matching. To further minimize confounding, we also compared treatments in increasingly more homogeneous population partitions, which is a novel approach that is different from most population studies comparing surgery and SBRT. Second, information on regional LNE provided by the National Cancer Database does not provide more details that may further enhance the quality of the study, such as which nodal stations were assessed, how many lymph nodes were taken from each nodal station, and whether endobronchial ultrasonography-guided mediastinal lymph node staging was done prior to surgery. These limitations, which can be overcome in randomized clinical trials, make our study more hypothesis generating than practice defining. However, our study does provide guidance for the design of future prospective trials and additional evidence for daily clinical decision-making in the lack of robust results from randomized clinical trials.

Only a small fraction of the patients underwent regional LNE of limited extent, or regional LN aspiration or biopsy in the SBRT cohort. These lymph node assessment procedures were not associated with any significant survival benefit in SBRT patients. This finding is also corroborated in previous studies.^36,37^ Together with our large sample size of predominantly patients receiving SBRT to the primary tumor only, their inclusion in the SBRT cohort most likely will not affect the results of our analyses or the generalizability of our study, which primarily pertains to patients who received treatment of the primary tumor only if they received SBRT, with additional regional lymph node assessment conducted only occasionally.

## Conclusions

Curative-intent surgery coupled with regional LNE, when both conducted to an appropriate extent, was associated with the best long-term OS for ES NSCLC. This makes it the preferred treatment for ES NSCLC. However, there may be situations in which SBRT could be a reasonable alternative treatment.

## Notes

Supplement. **eTable 1.** Other Patient Characteristics **eTable 2.** Overall Survival Based on Patient Characteristics **eTable 3.** Comparison of Long-term Survival After Surgery vs SBRT in Operable T1 Patients > 75 Years Old **eTable 4.** Propensity Score Matched Comparison of Long-term Survival After Sublobar Resection vs SBRT and Characteristics of Matched Patients **eTable 5.** Propensity Score Matched Comparison of Long-term Survival After Lobar Resection vs SBRT and Characteristics of Matched Patients **eFigure.** Average Adjusted HR for Mortality Risk After Surgery (Reference) vs SBRT From 16 Patient Population Partitions
