# Supplementary material for: Comparison of Long-term Survival of Patients With Early-Stage Non–Small Cell Lung Cancer After Surgery vs Stereotactic Body Radiotherapy
Source: JAMA Netw Open. 2019 Nov 20;2(11):e1915724. doi: 10.1001/jamanetworkopen.2019.15724 (PMC6902813; doi:10.1001/jamanetworkopen.2019.15724)
Supplement: Supplement. — eTable 1. Other Patient Characteristics eTable 2. Overall Survival Based on Patient Characteristics eTable 3. Comparison of Long-term Survival After Surgery vs SBRT in Operable T1 Patients > 75 Years Old eTable 4. Propensity Score Matched Comparison of Long-term Survival After Sublobar Resection vs SBRT and Characteristics of Matched Patients eTable 5. Propensity Score Matched Comparison of Long-term Survival After Lobar Resection vs SBRT and Characteristics of Matched Patients eFigure. Average Adjusted HR for Mortality Risk After Surgery (Reference) vs SBRT From 16 Patient Population Partitions [file jamanetwopen-2-e1915724-s001.pdf]

Supplementary Online Content

Chi A, Fang W, Sun Y, Wen S. Comparison of long-term survival of patients with early-stage non–small cell lung cancer after surgery vs stereotactic body radiotherapy. *JAMA Netw Open*. 2019;2(11):e1915724. doi:10.1001/jamanetworkopen.2019.15724

eTable 1. Other Patient Characteristics

eTable 2. Overall Survival Based on Patient Characteristics

eTable 3. Comparison of Long-term Survival After Surgery vs SBRT in Operable T1

Patients > 75 Years Old

eTable 4. Propensity Score Matched Comparison of Long-term Survival After Sublobar Resection vs SBRT and Characteristics of Matched Patients

eTable 5. Propensity Score Matched Comparison of Long-term Survival After Lobar Resection vs SBRT and Characteristics of Matched Patients

eFigure. Average Adjusted HR for Mortality Risk After Surgery (Reference) vs SBRT From 16 Patient Population Partitions

This supplementary material has been provided by the authors to give readers additional information about their work.

© 2019 Chi A et al. *JAMA Network Open.*

eTable 1. Other Patient Characteristics

	SBRT (n = 13,379)		Surgery (n = 91,330)	
**Characteristic**	**N (%)**		**N (%)**	***P***
**Insurance**				< 0.001
Uninsured	112 (0.8)		1,716 (1.9)	
Private	1,692 (12.7)		26,886 (29.4)	
Medicaid	521 (3.9)		4,472 (4.9)	
Medicare	10,419 (77.9)		56,305 (61.7)	
Other	635 (4.7)		1,951 (2.1)	
**Income quartile**				< 0.001
1	2,489 (18.6)		16,712 (18.3)	
2	3,494 (26.1)		22,238 (24.4)	
3	3,787 (28.3)		24,523 (26.9)	
4 (highest)	3,542 (26.5)		27,079 (29.7)	
**Education quartile**				< 0.001
1	2,027 (15.2)		14,666 (16.1)	
2	3,754 (28.1)		25,187 (27.6)	
3	4,640 (34.7)		30,764 (33.7)	
4 (highest)	2,897 (21.7)		19,977 (21.9)	
**Urban vs rural**				< 0.001
Metropolitan	10,662 (79.7)		72,365 (79.2)	
Nonmetroplitan	2,425 (18.1)		16,241 (17.8)	
Unknown	292 (2.2)		2,724 (3.0)	
**Facility location**				< 0.001
Northeast	2,626 (19.6)		19,104 (20.9)	
South Atlantic	2,977 (22.3)		21,772 (23.8)	
Central	6,215 (46.5)		39,266 (43.0)	
Mountain	460 (3.4)		3,081 (3.4)	
Pacific	1,101 (8.2)		8,107 (8.9)	
**Facility type**				< 0.001
Academic	5,493 (41.1)		30,730 (33.7)	
Nonacademic	7,886 (58.9)		60,600 (66.4)	
**Facility volume quartile**				< 0.001
1	728 (5.4)		7,848 (8.6)	
2	2,523 (18.9)		19,718 (21.6)	
3	5,239 (39.2)		32,293 (35.4)	
4 (highest)	4,889 (36.5)		31,471 (34.5)	
**Surgery at primary site**				
Wedge resection			13,377 (14.7)	
Segmentectomy			3,146 (3.4)	
Lobectomy			72,448 (79.3)	
Pneumonectomy			2,359 (2.6)	
**Systemic therapy**				

© 2019 Chi A et al. *JAMA Network Open.*

Chemotherapy				< 0.001
No	12,990 (97.1)		74,770 (81.9)	
Yes	389 (2.9)		16,560 (18.1)	
Immunotherapy				0.20
No	13,372 (100.0)		91,245 (99.9)	
Yes	7 (0.1)		85 (0.1)	
Both	6 (0.1)		59 (0.1)	

SBRT: Stereotactic body radiotherapy.

© 2019 Chi A et al. *JAMA Network Open.*

eTable 2. Overall Survival Based on Patient Characteristics

Characteristics	N	5-year OS	*P*
**Age**			< 0.001
≤ 65	38,083	68.9%	
66-70	20,508	61.5%	
71-75	19,745	56.1%	
76-80	15,337	48.5%	
>80	11,036	40.1%	
**Year of diagnosis**			< 0.001
2004-2009	28,704	56.3%	
2010-2015	76,005	60.6%	
**Sex**			< 0.001
Male	48,573	52.9%	
Female	56,136	64.3%	
**Race**			< 0.001
White	92,408	58.4%	
Black	8,603	61.9%	
Other	3,004	69.8%	
Unknown	694	63.4%	
**CCS**			< 0.001
0	51,087	63.4%	
1	36,786	58.0%	
≥ 2	16,836	47.9%	
**Insurance**			< 0.001
Uninsured	1,828	66.0%	
Private	28,578	70.0%	
Medicaid	4,993	57.5%	
Medicare	66,724	54.1%	
Other Government	1,442	53.8%	
**Income quartile**			< 0.001
1st	19,201	55.7%	
2nd	25,732	56.8%	
3rd	28,310	58.8%	
4th	30,621	64.0%	
**Education quartile**			< 0.001

© 2019 Chi A et al. *JAMA Network Open.*

1st	16,693	56.9%	
2nd	28,941	57.5%	
3rd	35,404	59.5%	
4th	22,874	62.8%	
**Tumor Location**			< 0.001
RUL	36,079	61.1%	
RML	5,031	58.7%	
RLL	18,641	57.6%	
LUL	27,310	58.5%	
LLL	14,719	58.6%	
Overlapping	968	48.3%	
Lung, NOS	1,461	51.6%	
Other	500	52.1%	
**Clinical T classification**			< 0.001
T1a	6,246	72.1%	
T1b	33,353	65.6%	
T1c	24,327	58.9%	
T2a	22,425	54.6%	
T2b	7,701	49.6%	
T3	10,370	47.7%	
**Tumor size (cm)**			< 0.001
≤ 3	72,582	62.9%	
> 3 – 5	24,612	51.2%	
> 5 – 7	7,420	47.9%	
**Histology**			< 0.001
Adenocarcinoma	64,592	64.6%	
Squamous cell carcinoma	32,502	51.3%	
Adenosquamous	2,641	48.1%	
Large cell carcinoma	1,222	50.2%	
Other	3,752	44.2%	
**Grade**			< 0.001
Well diff. NOS	15,327	74.7%	
Moderately diff.	44,174	60.5%	
Poorly diff./ Undifferentiated	33,327	53.8%	
**Tumor Eextension**			< 0.001
Confined to one lung	58,283	61.9%	
Adj. lobe ext.	225	55.9%	
MSB/Carina/Hilum	2,320	54.3%	
Other central structures	494	41.0%	
Pleura/chest wall/diaphragm	14,350	54.6%	
Atelect./ob.pneumonia	1,078	48.7%	

© 2019 Chi A et al. *JAMA Network Open.*

**Separate tumor nodules, ipsilateral lung**			< 0.001
None	72,014	61.0%	
Yes, same lobe	2,919	55.8%	
Yes, different lobe involved	0	0.0%	
Separate nodules, NOS	0	0.0%	
**Scope of LN surgery**			< 0.001
No	20,187	39.1%	
Yes	84,172	63.4%	
**LN. examined**			< 0.001
0	19,620	39.5%	
1-5	25,919	61.0%	
6-10	25,429	64.4%	
11-15	14,273	65.3%	
16-20	6,726	65.0%	
21-25	2,981	64.4%	
26-90	3,251	63.8%	
Aspiration	700	42.9%	
NOS	5,628	62.5%	
**Primary Treatment**			< 0.001
SBRT	13,379	30.4%	
Wedge resection	13,377	53.3%	
Segmentectomy	3,146	59.4%	
Lobectomy	72,448	64.6%	
Pneumonectomy	2,359	48.1%	
**Systemic therapy**			
Chemotherapy			0.95
No	87,760	59.2%	
Yes	16,949	57.9%	
Immunotherapy			0.14
No	104,617	59.0%	
Yes	92	62.2%	
**Urban/Rural**			< 0.001
Metropolitan	83,027	60.3%	
Urban	16,357	54.4%	
Rural	2,309	54.0%	
**Facility type**			< 0.001
Academic	36,223	61.9%	
Nonacademic	68,486	57.5%	
**Facility Location**			< 0.001
Northeast	21,730	63.0%	

© 2019 Chi A et al. *JAMA Network Open.*

South Atlantic	24,749	59.1%	
Central	45,481	56.6%	
Mountain	3,541	59.4%	
Pacific	9,208	61.4%	
**Facility volume quartile**			< 0.001
1st	8,417	56.8%	
2nd	23,403	57.8%	
3rd	36,883	59.9%	
4th	36,006	59.7%	
**Pathological LN positivity**			< 0.001
0	73,801	65.9%	
1 – 2	7,528	47.9%	
3 – 6	2,693	41.3%	
7 – 10	419	31.8%	
> 10	126	20.3%	
# NOS	249	42.6%	
None examined	19,620	39.5%	
Unknown	266	54.4%	
**Pathological margin status**			< 0.001
(-)	86,920	63.4%	
(X)	227	41.8%	
(+), Microscopic	1,991	38.0%	
(+), Macroscopic	135	31.2%	
(+), NOS	1,281	41.2%	
Unknown	776	51.5%	

OS: Overall survival; CCS: Charlson Comorbidity Score.; RUL: Right upper lobe; RML: Right middle lobe; RLL: Right lower lobe; LUL: Left upper lobe; LLL: Left lower lobe; NOS: Not otherwise specified; diff.: differentiated; Adj.: Adjacent; Ext.: extension; MSB: mainstem bronchus; Atelect.: atelectasis; Ob.: Obstructive; LN: lymph node(s). (-): negative; (+): positive; (X): Indeterminate.

© 2019 Chi A et al. *JAMA Network Open.*

eTable 3. Comparison of Long-term Survival after Surgery vs SBRT in Operable T1 Patients > 75 Years Old

Study Variables	N	HR*	95% CI	*P*
**Treatment**				
SBRT	89	Reference		
Sublobar resection	1,571	1.17	0.64 - 2.15	0.60
Lobectomy	3,796	1.07	0.57 - 2.00	0.84
Pneumonectomy	29	3.20	1.20 - 8.49	0.02
**Histology**				
Adenocarcinoma	3,858	Reference		
Squamous cell carcinoma	1,313	1.31	1.08 - 1.57	0.005
Other	314	1.84	1.31 - 2.60	0.001
**Tumor extension**				
Confined to the lung	3,541	Reference		
Others	566	1.30	1.07 - 1.59	0.009
**Separate tumor nodules**				
No	3,638	Reference		
Yes	139	1.88	1.30 - 2.71	0.001

*Also adjusted for the number of lymph nodes examined. HR: Hazard Ratio for mortality risk; SBRT: Stereotactic body radiotherapy.

© 2019 Chi A et al. *JAMA Network Open.*

eTable 4. Propensity Score Matched Comparison of Long-term Survival after Sublobar Resection vs SBRT and Characteristics of Matched Patients

**Survival Comparison**	**HR (95% CI)**	***P***
Sublobar Resection (n =12,632) vs SBRT (n =12,632)	0.56 (0.54, 0.58)	<0.001
**Based on #LN Examined During Surgery**		
Sublobar Resection (0) vs SBRT	0.68 (0.64, 0.71)	<0.001
Sublobar Resection (1 - 10) vs SBRT	0.51 (0.49, 0.54)	<0.001
Sublobar Resection (11 - 15) vs SBRT	0.42 (0.36, 0.48)	<0.001
Sublobar Resection (> 15) vs SBRT	0.33 (0.27, 0.41)	<0.001
**Characteristics of Matched Patients**	**Sublobar Resection (n = 12,632)**	**SBRT (n = 12,632)**
	**N (%)**	**N (%)**
**Age**		
≤ 75	8,303 (66)	6,526 (52)
>75	4,329 (34)	6,106 (48)
**Sex**		
Male	5,512 (44)	5,691 (45)
Female	7,120 (56)	6,941 (55)
**Tumor grade**		
1	2,157 (17)	1,021 (8)
2	5,314 (42)	2,316 (18)
3 or 4	4,145 (33)	2,675 (21)
Unknown	1,016 (8)	6,620 (52)
**Clinical T stage**		
T1	9,370 (74)	8,980 (71)
T2	2,643 (21)	3,050 (24)
T3	619 (5)	602 (5)
**Race**		
White	11,303 (89)	11,239 (89)
Black	981 (8)	1,097 (9)

© 2019 Chi A et al. *JAMA Network Open.*

Others	256 (2)	206 (2)
Unknown	92 (1)	90 (1)
**Charlson Comorbidity Score**		
0	5,784 (46)	6,609 (52)
1	4,302 (34)	3,598 (28)
2 or 3	2,546 (20)	2,425 (19)
**Histology**		
Adenocarcinoma	7,656 (61)	6,176 (49)
Squamous cell carcinoma	4,230 (33)	4,815 (38)
Adenosquamous	181 (1)	127 (1)
Large cell carcinoma	128 (1)	75 (1)
Other	437 (3)	1,439 (11)

HR: Hazard Ratio for mortality risk; CI: Confidence Interval; SBRT: Stereotactic body radiotherapy.

© 2019 Chi A et al. *JAMA Network Open.*

eTable 5. Propensity Score Matched Comparison of Long-term Survival after Lobar Resection vs SBRT and Characteristics of Matched Patients

**Survival Comparison**	**HR (95% CI)**	***P***
Lobar Resection (n = 12,632) vs SBRT (n = 12,632)	0.47 (0.45, 0.49)	< 0.001
**Based on #LN Examined During Surgery**		
Lobar Resection (0) vs SBRT	0.54 (0.47, 0.62)	< 0.001
Lobar Resection (1 - 10) vs SBRT	0.46 (0.44, 0.48)	< 0.001
Lobar Resection (11 - 15) vs SBRT	0.46 (0.42, 0.49)	< 0.001
Lobar Resection (> 15) vs SBRT	0.46 (0.43, 0.51)	< 0.001
**Characteristics of Matched Patients**	**Lobar Resection (n = 12,632)**	**SBRT (n = 12,632)**
	**N (%)**	**N (%)**
**Age**		
≤ 75	5,929 (47)	6,526 (52)
>75	6,703 (53)	6,106 (48)
**Sex**		
Male	5,722 (45)	5,691 (45)
Female	6,910 (55)	6,941 (55)
**Tumor grade**		
1	1,863 (15)	1,021 (8)
2	3,220 (25)	2,316 (18)
3 or 4	4,236 (34)	2,675 (21)
Unknown	3,313 (26)	6,620 (52)
**Clinical T stage**		
T1	8,652 (68)	8,980 (71)
T2	3,262 (26)	3,050 (24)
T3	718 (6)	602 (5)
**Race**		

© 2019 Chi A et al. *JAMA Network Open.*

White	11,309 (90)	11,239 (89)
Black	1,026 (8)	1,097 (9)
Others	210 (2)	206 (2)
Unknown	87 (1)	90 (1)
**Charlson Comorbidity Score**		
0	6,462 (51)	6,609 (52)
1	3,847 (30)	3,598 (28)
2 or 3	2,323 (18)	2,425 (19)
**Histology**		
Adenocarcinoma	6,410 (51)	6,176 (49)
Squamous cell carcinoma	5,036 (40)	4,815 (38)
Adenosquamous	167 (1)	127 (1)
Large cell carcinoma	85 (1)	75 (1)
Other	934 (7)	1,439 (11)

HR: Hazard Ratio; CI: Confidence Interval; SBRT: Stereotactic body radiotherapy.

© 2019 Chi A et al. *JAMA Network Open.*

eFigure. Average Adjusted HR for Mortality Risk after Surgery (Reference) vs SBRT from 16 Patient Population Partitions

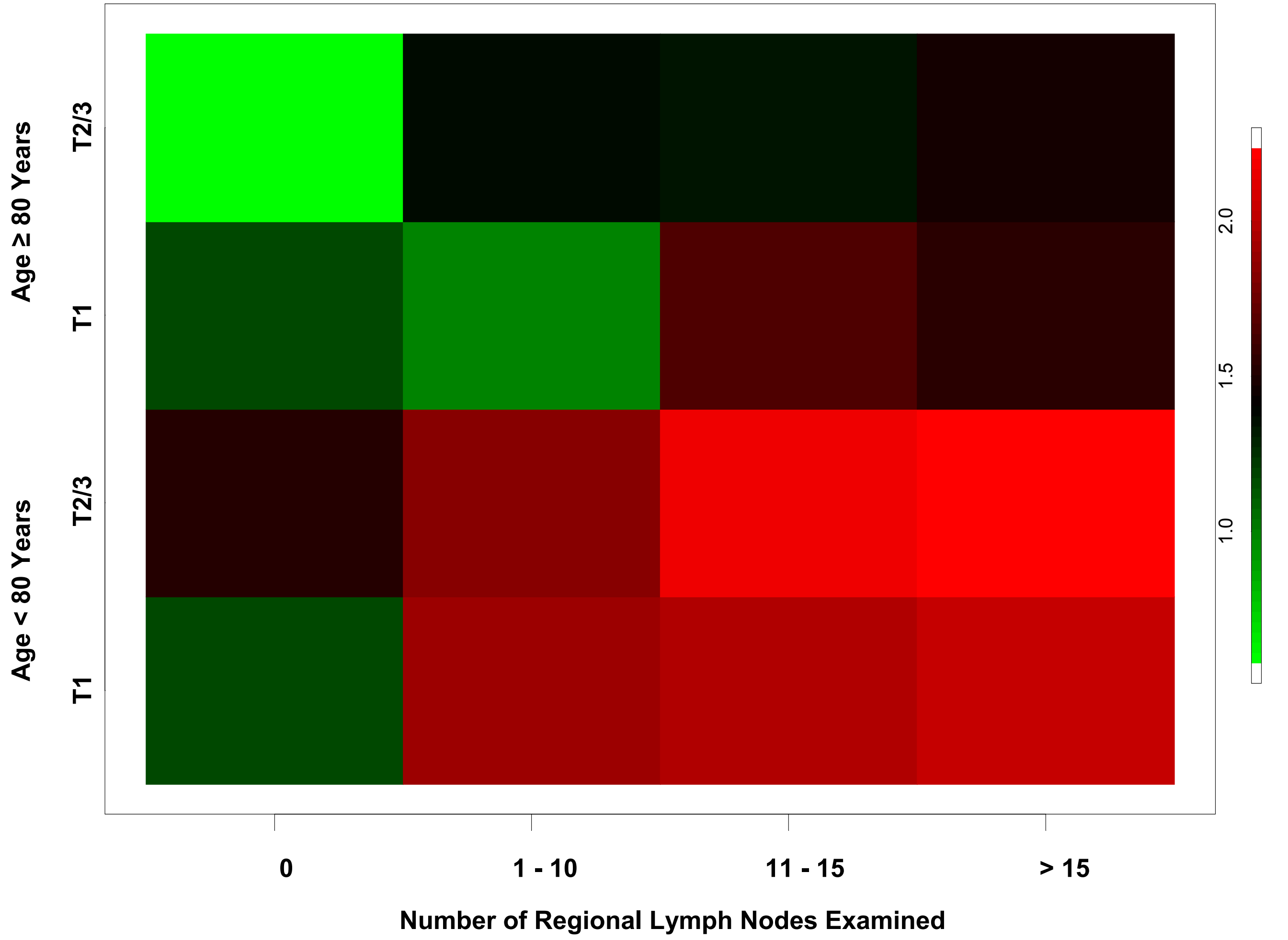

© 2019 Chi A et al. *JAMA Network Open.*
